# *Candida* spp.: the burden of a microorganism in a microbiology department

**DOI:** 10.1128/spectrum.03860-23

**Published:** 2024-07-09

**Authors:** Ana Soriano-Martín, Roberto Alonso, Marina Machado, Elena Reigadas, Patricia Muñoz, Emilio Bouza

**Affiliations:** 1 Clinical Microbiology and Infectious Diseases Department, Hospital General Universitario Gregorio Marañón, Madrid, Spain; 2 Instituto de Investigación Sanitaria Gregorio Marañón, Madrid, Spain; 3 Medicine Department, School of Medicine, Universidad Complutense de Madrid, Madrid, Spain; 4 CIBER Enfermedades Respiratorias-CIBERES (CB06/06/0058), Madrid, Spain; Houston Methodist Hospital, Houston, Texas, USA

**Keywords:** *Candida* burden, *Candida* cultures, clinical microbiology department, laboratory workload, costs

## Abstract

There is no precise information available on the entire workload of isolating a specific microorganism in a clinical microbiology laboratory, and the costs associated with it have not been specifically estimated. In this descriptive retrospective study conducted at the microbiology department of a general teaching hospital from January 2021 to December 2022, we assessed the workload associated with identifying *Candida* species in all types of clinical samples and patients. Costs were estimated from data obtained from the hospital’s finance department and microbiology laboratory cost records. In 2 years, 1,008,231 samples were processed at our microbiology department, of which 8,775 had one or more *Candida* spp. isolates (9,683 total isolates). Overall, 5,151 samples with *Candida* spp. were identified from 2,383 inpatients. We isolated *Candida* spp. from 515.3 samples/100,000 population/year and from 92 samples/1,000 hospital admissions/year. By sample type, 90.8% were superficial, mainly mucosal. Only 9.1% *Candida* spp. were isolated from deep, usually sterile, samples, being mostly from ordinarily sterile fluids. *Candida albicans* was the main species (58.5%) identified, followed by *C. parapsilosis* complex, *C. glabrata*, *C. tropicalis,* and *C. krusei*. In admitted patients, the incidences of samples with *Candida* spp. isolates were 302.7 samples/100,000 population/year and 54 samples/1,000 admissions/year. The average cost of isolating and identifying *Candida* spp. was estimated at 25€ per culture-positive sample. To our knowledge, this is the first attempt to gage the workload and costs of *Candida* spp. isolation at a hospital microbiology department. These data can help assess the burden and significance of *Candida* isolation at other institutions and also help design measures for streamlining.

**IMPORTANCE:**

We believe that this work is of interest because at present, there is no really accurate information available on the total workload involved in isolating a specific microorganism in a clinical microbiology laboratory. The costs related to this have also not been described. We have described the unrestricted workload of Candida spp. in all types of samples for all types of species and patients. We believe that this information would be necessary to collect and share this information as well as to collect it in a standardized way to know the current situation of Candida spp. workload in all clinical microbiology laboratories.

## INTRODUCTION

Yeast of the genus *Candida* colonize or invade superficial or deep human tissues in individuals with very different underlying diseases, different clinical syndromes, and highly variable prognosis (1, 2). The information on the workload that is represented by isolation and identification of *Candida* spp. for the human health system has been only selectively estimated, for special populations of patients, particular clinical syndromes and given species of the genus. However, a global perception of the burden of *Candida* spp. for microbiology departments is lacking. Most of the studies carried out deal with colonization by *Candida* spp. mainly developed in intensive care units (ICUs), neonatology units, and pediatric services (3–10). We were unable to find studies estimating the overall burden of *Candida* isolation and identification in microbiology departments. Moreover, studies on costs attributable to isolation and identification of specific microorganisms have been very rare and imprecise (11–15).

The aim of the present study was to assess the workload associated with isolating and identifying *Candida* spp. in a hospital microbiology department and to estimate the related costs.

## MATERIALS AND METHODS

### Study design, population, and setting

This was a descriptive retrospective study carried out at the Hospital General Universitario Gregorio Marañón (Madrid, Spain), a public hospital with 1,087 beds. This general and referral hospital serves an area with 851,057 inhabitants (data from the latest reports of the National Institute of Statistics revised in June 2023). Our microbiology department processes an average of 500,000 clinical samples per year. Our study was carried out from January 2021 to December 2022, and we used data from electronic HCIS software (Health Care Information System) and the microbiology department’s sample registration system according to a pre-established data collection form.

Variables recorded retrospectively for analysis included medical record number, patient initials, gender, requesting service of samples, service categorized into different medical departments (ICU, surgical, and emergency), bed number, sample number, sample registration date, sample type, classification by sample type and subgroups, and identified *Candida* species.

### Sample source

Samples were classified as superficial and deep. Superficial samples were defined as those collected from tissues or fluids that are not normally sterile and classified into cutaneous samples (fine needle aspiration, biopsies, eschar, skin lesion, hair, etc.), mucosal samples (bronchoaspirate, bronchoalveolar lavage, sputum, and several exudates such as otic, urethral, vaginal, etc.), catheters (including also catheter hubs and blood drawn by catheter lumens), and urine samples (including those obtained from the catheter).

Deep samples were those collected from usually sterile internal tissues and organs through sterile procedures. These samples were classified into ordinarily sterile tissues (liver abscesses, lymph nodes, bone, pericardial, pleural, lung biopsies, etc.), ordinarily sterile fluids (peritoneal, pleural, biliary, cerebrospinal, etc.), and blood.

### Samples by patient

Patients were classified into inpatients (defined as individuals who were admitted to hospital for at least 24 hours when sampled) and outpatients.

### Origin of samples by hospital wards

Hospital wards submitting samples for microbiological diagnosis were classified as medical, ICU, surgical, and emergency.

### Assessment of laboratory samples

Laboratory workload was reported as samples and isolates expressed as the denominator per 1,000 admissions/year and 100,000 inhabitants/year.

### *Candida* spp. identification

Routinely, samples with *Candida* spp. are processed and identified according to the standards and working procedure of the hospital’s microbiology laboratory, which is based on the clinical microbiology procedures of the Spanish Society of Infectious Diseases and Clinical Microbiology (SEIMC) (16, 17).

### Economic burden

To estimate the costs of isolating *Candida* spp. at our large institution, data were acquired from the hospital’s finance department and cost records of the microbiology laboratory. Costs were grouped into laboratory costs (including costs of equipment and reagents and anti-fungal drug sensitivity testing) and manpower costs (doctors, technicians, nurses, administrative staff, etc.). For the evaluation of the economic burden, costs were calculated as laboratory and staff costs corresponding to the percentage of workload associated with processing samples with *Candida* spp. in relation to the total number of samples processed. We then calculated the average cost per positive sample and isolate processed.

### Statistical analysis

Data were analyzed exclusively as proportions using the descriptive analysis tools of the statistical software package IBM SPSS 28.0.0.0.0.

## RESULTS

### Overall results of the *Candida* spp. workload

Over the 2-year study period (2021–2022), our center served a population of approximately 851,000 inhabitants and had 95,192 hospital admissions. The microbiology department processed 1,008,231 samples, 573,794 in 2021 and 434,437 in 2022. Of all the samples processed, 8,775 had one or more *Candida* spp. isolates, with a total of 9,683 *Candida* spp. isolates corresponding to 5,167 different patients.

**Fig 1:**
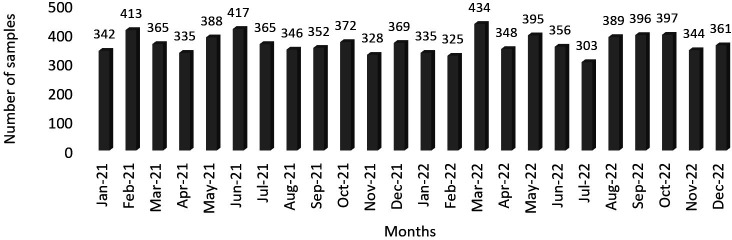
Number of samples with *Candida* spp. received and processed by the laboratory by months.

In Table 1, we provide data reflecting the workload associated with *Candida* during the study period. Accordingly, 0.87% of the samples tested had one or more *Candida* spp. isolates. The samples with *Candida* isolates per 100,000 population/year and per 1,000 hospital admissions/year were, respectively, 515.3 and 92.2. As shown in Fig. 1, the distribution of *Candida* spp. samples processed per month was uniform.

**TABLE 1:** Workload associated with *Candida* spp. expressed as numbers of samples, isolates, or patients with *Candida* spp. per 1,000 admissions/year and per 100,000 population/year

	1,000 admissions/yr	100,000 inhab/yr	Total N
Samples with *Candida* spp.	92.2	515.3	8,775
*Candida* spp. isolates	101.7	568.9	9,683
Patients with *Candida* spp. in one or more samples (overall)	54.3	303.5	5,167
Hospitalized patients with *Candida* spp.	25.0	140.0	2,383
Samples from hospitalized patients with *Candida* spp. (overall)	54.1	302.7	5,153

### Results of sample classification and isolates with *Candida* spp

Samples with *Candida* spp. were divided into different groups and subgroups, as shown in Table 2. Among these samples, 90.8% of them were superficial, 9.1% were deep, and the rest were unclassified. Of the superficial samples, the majority (70.8%) were mucosal samples, 16.5% were skin samples (1,313), 10.9% were urine samples (867), and 1.8% were catheter samples (144). Regarding deep samples, 43.6% (347) belonged to ordinarily sterile liquids, 32% (255) to ordinarily sterile tissues, and 24.4% (194) to blood. In the total number of deep samples processed (796), 83 episodes of candidemia were detected in 82 patients. A total of 82 episodes correspond to 81 inpatients and one to non-hospitalized patient. The number of patients with invasive candidiasis different from candidemia was 339.

**TABLE 2:** Workload associated with *Candida* spp. over the total number of samples and isolates received and processed in the Department of Clinical Microbiology, expressed in absolute values and incidences of samples per 1,000 admissions/year and per 100,000 population/year^*a*^

	Total N (%)	1,000 admissions/yr	100,000 inhab/yr
Superficial samples	7,969 (90.8)	83.7	468.2
Mucosal Skin Urine Catheters^*b*^	5,642 (70.8) 1,313 (16.5) 867 (10.9) 144 (1.8)	59.3 13.8 9.1 1.5	331.5 77.1 50.9 8.5
Catheter tips only	83 (1)	0.9	4.9
Deep samples	796 (9.1)	8.4	46.8
Ordinarily sterile fluids	347 (43.6)	3.6	20.4
Ordinarily sterile tissues	255 (32.0)	2.7	15.0
Blood	194 (24.4)	2.0	11.4
Other samples (not classified)	10 (0.1)	0.1	0.6
Samples with *Candida* spp. from inpatients Samples with *Candida* spp. from outpatients	5,153 (58.7) 3,622 (41.3)	54.1 38.1	302.7 212.8
Samples from non-hospital services Samples from intrahospital services	1,812 (20.6) 6,959 (79.3)	19 73	106.5 408.8
Medical wards ICU Surgical wards Emergency department	3,499 (50.3) 1,728 (24.8) 1,170 (16.8) 558 (8.0)	36.8 18.2 12.3 5.9	205.6 104.7 68.7 32.8
Not reported	4 (0.05)	0.04	0.2
*Candida* spp. isolates	9,683	101.7	568.9
*Candida albicans*	5,668 (58.5)	59.5	666.0
*Candida parapsilosis, metapsilosis,* and *orthopsilosis*	1,704 (17.6)	28.8	100.1
*Candida glabrata*	1,068 (11.0)	11.2	62.7
*Candida tropicalis*	587 (6.1)	6.2	34.5
*Candida krusei*	232 (2.4)	2.4	13.6
Candida auris^*c*^	1 (0.01)	0.01	0.06
Others	423 (4.4)	4.4	24.9
*Candida lusitaniae* *Candida guilliermondii* *Candida dubliniensis* *Candida kefyr* *Candida rugosa and pararugosa* *Candida lipolytica* *Candida famata* *Candida intermedia* *Candida sp*. *Candida bracarensis* *Candida lambica* *Candida zeylanoides* *Candida inconspicua* *Candida boidinii* *Candida lactis-condensi* *Candida sake* *Candida haemulonii*	119 (1.2) 92 (1.0) 77 (0.8) 43 (0.4) 24 (0.2) 19 (0.2) 13 (0.1) 11 (0.1) 6 (0.1) 5 (0.1) 5 (0.1) 3 (0.03) 2 (0.02) 1 (0.01) 1 (0.01) 1 (0.01) 1 (0.01)	1.3 1.0 0.8 0.5 0.3 0.2 0.1 0.1 0.1 0.1 0.1 0.03 0.02 0.01 0.01 0.01 0.01	7.0 5.4 4.5 2.5 1.4 1.1 0.8 0.6 0.4 0.3 0.3 0.2 0.1 0.06 0.06 0.06 0.06

^*a*^ Abbreviations: ICU, intensive care unit.

^*b*^ Catheters: including catheter tips, catheter hubs, and blood from the catheter lumen.

^*c*^ From a quality control sample submitted by the Spanish Society of Infectious Diseases and Clinical Microbiology (SEIMC).

Most of the samples came from intrahospital units (79.3%), whereby half of them were from medical wards followed by the ICU. Of the samples from non-hospital services, almost 100% were superficial, mainly mucosal (71%) and cutaneous (27%).

The main *Candida* species isolated was *Candida albicans* (58.5%), followed by *C. parapsilosis* complex (*C. parapsilosis, C. metapsilosis,* and *C. orthopsilosis*) (17.6%), *C. glabrata* (11%), *C. tropicalis* (6.1%), and *C. krusei* (2.4%). The remaining isolates were identified as other species, which were found in lower proportions, as shown in Table 2.

### Total results of inpatients with *Candida* spp

Over the 2-year study period, *Candida* spp. isolates (one or more) were detected in 2,383 inpatients. In total, 5,153 samples were registered, and 5,807 isolates were identified in these inpatients. Table 3 details all information associated with samples from these patients. The number of samples with *Candida* spp. isolates in inpatients per 100,000 population/year and per 1,000 hospital admissions/year was 302.7 and 54.1, respectively.

**TABLE 3:** Workload associated with *Candida* spp. from samples and isolates from inpatients, received and processed in the Department of Clinical Microbiology, expressed in absolute values and incidence of samples per 1,000 admissions/year and per 100,000 population/year^*a*^

	Total N (%)	1,000 admissions/yr	100,000 Inhab/yr
Inpatients with *Candida* spp.	2,383	25.0	140.0
Samples from inpatients Gender: male	5,153 3,173 (61.6)	54.1 33.3	302.7 186.4
Superficial samples	4,488 (87.1)	47.1	263.7
Mucosal Urine Skin Catheters^*b*^	3,264 (72.7) 721 (16.1) 367 (8.2) 136 (3.0)	34.3 7.6 3.9 1.4	191.8 84.7 21.6 8.0
Catheter tips only	76 (1.7)	0.8	4.5
Deep samples	657 (12.7)	6.9	38.6
Ordinarily sterile fluids Ordinarily sterile tissues Blood	264 (40.3) 204 (31.1) 188 (28.6)	2.8 2.1 2.0	15.5 12.0 11.0
Other samples (not classified)	8 (0.2)	0.1	0.9
Medical services	2,054 (39.9)	21.6	120.7
Medical ward ICU Surgical ward Emergency department	1,751 (34) 1,186 (23) 162 (3.1)	18.4 12.5 1.7	102.9 69.7 9.5
*Candida* spp. isolates from inpatient samples	5,807	61.0	341.2
*Candida albicans*	3,357 (57.8)	35.3	197.2
*Candida parapsilosis, metapsilosis* and *orthopsilosis*	767 (30.4)	8.1	45.0
*Candida glabrata*	759 (13.2)	8.0	44.6
*Candida tropicalis*	479 (8.3)	5.03	28.1
*Candida krusei*	169 (2.9)	2.8	9.9
Others	267 (4.6)	2.8	15.6
*Candida lusitaniae* *Candida dubliniensis* *C. guilliermondii* *Candida kefyr* *Candida rugosa y pararugosa* *Candida lipolytica* *Candida lambica* *Candida bracarensis* *Candida intermedia* *Candida sp*. *Candida zeylanoides*	97 (1.7) 71 (1.2) 36 (0.6) 34 (0.6) 20 (0.3) 4 (0.07) 4 (0.07) 2 (0.03) 2 (0.03) 2 (0.03) 2 (0.03)	1.0 0.7 0.4 0.4 0.2 0.04 0.04 0.02 0.02 0.02 0.02	6.7 4.2 2.1 2.0 1.2 0.2 0.2 0.1 0.1 0.1 0.1

^*a*^ Abbreviations: ICU, intensive care unit.

^*b*^ Catheters: including catheter tips, catheter hub cultures, and blood from the catheter lumens.

Samples from the inpatients were mostly superficial (87.1%), mainly mucosal samples. Of the deep samples, most belonged to ordinarily sterile fluids. According to microbiological profiles, species distributions were as described previously for both out- and inpatients. *C. albicans* was the most commonly isolated species, followed by the *C. parapsilosis* complex among the non-*albicans* species.

Of the inpatients (2,383), 2,188 had superficial infection and 351 deep infection. Eighty-one of these inpatients had 82 episodes of candidemia.

### Results of the relationship between *Candida* species and inpatient sample type

Table 4 shows the proportions of species from inpatients identified in the different types of samples, both superficial and deep. In all sample types, *C. albicans* was the most frequent species. Among the non-*albicans* species, the *C. parapsilosis* complex was the most common in cutaneous, catheter, and blood samples. *C. glabrata* was mainly found in urine, followed by mucosal and sterile tissue samples. Both these species appeared in similar proportions in sterile fluids.

**TABLE 4:** Distribution of *Candida* spp. in different samples from hospitalized patients

*Candida* species	Superficial samples				
	Cutaneous N (%)	Mucosal samples N (%)	Catheter N (%)	Urine N (%)	TOTAL N
*Candida albicans*	238 (61.5)	2,333 (61.2)	50 (36.8)	358 (48.4)	2,979
*Candida parapsilosis, metapsilosis,* and *orthopsilosis*	87 (22.5)	436 (11.4)	58 (42.6)	63 (8.5)	644
*Candida glabrata*	26 (6.7)	441 (11.6)	7 (5.1)	184 (24.9)	658
*Candida tropicalis*	21 (5.4)	271 (7.1)	17 (12.5)	98 (13.2)	407
*Candida krusei*	5 (1.3)	139 (3.6)	1 (0.7)	8 (1.1)	153
Others	10 (2.6)	194 (5.1)	3 (2.2)	29 (3.9)	236
TOTAL	389	3814	136	740	5077

*Candida* species	Deep samples							
	Ordinarily sterile tissues N (%)		Ordinarily sterile fluids N (%)		Blood N (%)		TOTAL N	
*Candida albicans*	129 (54.9)		154 (51.7)		89 (47.1)		372	
*Candida parapsilosis, metapsilosis,* and *orthopsilosis*	34 (14.5)		40 (13.4)		49 (25.9)		123	
*Candida glabrata*	37 (15.7)		40 (13.4)		22 (11.6)		99	
*Candida tropicalis*	13 (5.5)		33 (11.1)		26 (13.8)		72	
*Candida krusei*	7 (3.0)		7 (2.3)		2 (1.1)		16	
Others	15 (6.4)		24 (8.1)		1 (0.5)		40	
TOTAL	235		298		189		722	

### Results of the cost estimation associated with *Candida* spp

We estimated the present costs of isolating *Candida* spp. at a large institution. Costs related to the microbiology service are shown in Table 5. Samples with *Candida* spp. represent 0.87% of the workload in relation to the total number of samples processed at the laboratory. Accordingly, the average cost per sample was estimated at 25€ (approximately 109,687.5 €/year). In 8,775 samples found to have *Candida* species, 9,683 isolates were identified; hence, one isolate would correspond to approximately 0.9 samples. This indicates that the costs needed to identify one isolate were similar to the costs for processing a sample, that is, 23.6€.

**TABLE 5:** Estimate and distribution of costs from the microbiology laboratory over the 2-year study period

Year	2021	2022	Total
Total costs of the microbiology laboratory finance department (€)	7,746,882	6,282,884	14,029,766
Microbiology laboratory finance department costs for total *Candida* spp. samples (€)			122,058.96
Total costs of microbiology department staff (€)	5,890,421	5,458,227	11,348,648
Costs of microbiology department staff for samples with *Candida* spp.			98,733.24
Laboratory and staff costs equivalent to the work represented by samples with *Candida* spp.			220,792.2
Estimated cost per sample with *Candida* spp. (€) Estimated cost per *Candida* spp. isolate (€)			25.2 23.6

## DISCUSSION

In our general hospital, *Candida* species are isolated in approximately 1% of all samples received in the microbiology laboratory. This represents 515.3 samples/100,000 population/year and 92 samples/1,000 hospital admissions/year. We estimated a cost of 25€ for each of the samples in which *Candida* spp. is isolated.

Few studies have assessed the workload associated with the identification of specific microorganisms (18–24), although there are some reports describing the laboratory workload related to *Streptococcus pneumoniae* and *Aspergillus fumigatus* (25, 26). In the case of *Candida* spp., workload data have been overall biased, and the information available refers almost exclusively to the workload associated with candidemia (27–35). As far as we know, no literature information exists regarding the overall workload of all *Candida* spp. isolates.

Our work shows that a high percentage of clinical samples showing *Candida* spp. came from hospitalized patients, yet over 80% of isolates did not correspond to deep samples. At our center, only 3.4% of all inpatients in whom one or more *Candida* spp. were isolated from any sample type had candidemia. However, if we selected cases with *Candida* spp. isolated from deep samples, 23.08% were candidemic. This means the incidence of candidemia under-reflects the total burden of cases with *Candida* spp. isolates admitted to a general hospital and also underestimates the episodes of invasive candidiasis. Among the samples collected from non-hospitalized patients, episodes of invasive or deep candidiasis were extraordinarily rare.

Of all the *Candida* species detected, we confirmed that *C. albicans* remains the most frequently isolated species (representing more than 50% of all species), considering invasive samples and also all types of samples from inside and outside the hospital (36). We did not identify any strain of *Candida auris* in our institution, despite it recently becoming a threatening species since its first appearance in 2009 (37). *Candida auris* is, nevertheless, known to show an irregular distribution pattern (38).

Patients colonized or infected with *Candida* spp. were not only ICU patients but also hospitalized patients in both medical and surgical units, as described in the literature (39, 40). As per our study results, patients are mainly from medical wards (approximately 50% of all samples with *Candida* spp.), followed by the ICU and surgical wards.

Invasive fungal infections (IFI) are now known to generate high morbidity and mortality, along with a high associated economic burden, as indicated by the review data reported in 2014 by Drgona *et al*. (41). Several reports have presented hospital costs for different diseases. However, we found no study focusing on determining the costs of isolation, identification, and drug sensitivity testing from a clinical microbiology laboratory, rather than the clinical and hospitalization perspectives as, for example, the study by Slavin (35, 42–49).

Authors such as Brezmes *et al*. (50) did effectively estimate the cost of reagents, staff, and working times for each test performed in the clinical laboratory. They estimated an average cost of 16.05€ per test. As expected, they found large cost differences between positive and negative tests. In another example, Kaplan *et al.* determined an average cost of 34.50$ per urine culture, including identification and drug sensitivity testing (51).

Our cost estimates of of approximately 25€ per sample for isolating and identifying *Candida* spp. in a microbiology department could serve as a reference for healthcare economist and laboratory directors. The importance of efficient resource allocation and targeted interventions to reduce the impact of *Candida* spp. on healthcare expenditures and patient care costs should be underscored.

### Limitations

The main limitations of this study were that it is a single-center study and that information was collected retrospectively. However, we should stress that the literature lacks similar information.

Our work attempts to provide a broader view of the burden of *Candida* for microbiology departments, which would put the quantitative and proportional importance of this yeast into a better perspective.
